# The Sociospatial Network: Risk and the Role of Place in the Transmission of Infectious Diseases

**DOI:** 10.1371/journal.pone.0146915

**Published:** 2016-02-03

**Authors:** James J. Logan, Ann M. Jolly, Justine I. Blanford

**Affiliations:** 1 School of Epidemiology, Public Health and Preventative Medicine, University of Ottawa, Ottawa, Ontario, Canada; 2 Department of Geography, Dutton Institute of e-Education and GeoVISTA Center, The Pennsylvania State University, University Park, Pennsylvania, United States of America; Burnet Institute, AUSTRALIA

## Abstract

Control of sexually transmitted infections and blood-borne pathogens is challenging due to their presence in groups exhibiting complex social interactions. In particular, sharing injection drug use equipment and selling sex (prostitution) puts people at high risk. Previous work examining the involvement of risk behaviours in social networks has suggested that social and geographic distance of persons within a group contributes to these pathogens’ endemicity. In this study, we examine the role of place in the connectedness of street people, selected by respondent driven sampling, in the transmission of blood-borne and sexually transmitted pathogens. A sample of 600 injection drug users, men who have sex with men, street youth and homeless people were recruited in Winnipeg, Canada from January to December, 2009. The residences of participants and those of their social connections were linked to each other and to locations where they engaged in risk activity. Survey responses identified 101 unique sites where respondents participated in injection drug use or sex transactions. Risk sites and respondents’ residences were geocoded, with residence representing the individuals. The sociospatial network and estimations of geographic areas most likely to be frequented were mapped with network graphs and spatially using a Geographic Information System (GIS). The network with the most nodes connected 7.7% of respondents; consideration of the sociospatial network increased this to 49.7%. The mean distance between any two locations in the network was within 3.5 kilometres. Kernel density estimation revealed key activity spaces where the five largest networks overlapped. Here, the combination of spatial and social entities in network analysis defines the overlap of vulnerable populations in risk space, over and above the person to person links. Implications of this work are far reaching, not just for understanding transmission dynamics of sexually transmitted infections by identifying activity “hotspots” and their intersection with each social network, but also for the spread of other diseases (e.g. tuberculosis) and targeting prevention services.

## Introduction

Sexually transmitted infections (STIs; such as chlamydia, gonorrhea, and infectious syphilis) and blood-borne pathogens (BBPs; specifically HIV and Hepatitis C) are treatable diseases that are concentrated in very small segments of our populations [1–4]. Each year, 448 million new cases of curable STIs occur in adults throughout the world and many live unaware that they are infected [5,6]. STIs and BBPs require close contact for transmission [7]. They are diseases with a significant basis in behavioural patterns; their spread through a population occurs neither uniformly nor randomly [8]. Rather, it is often a function of complex, often intimate and/or taboo social interactions between two individuals or a network of people. Epidemiologically, emphasis is placed on populations engaging in risk-based behaviours like unprotected sex, sex with many partners, and use of injected drugs through needle sharing [8,9].

In order to more accurately define patterns of BBP and STI epidemiology, both known and unidentified links between people are required to delineate exposure and transmission. Infection and recurrent infection in these social groups suggests that proximity is a significant factor in the endemic propagation of these pathogens. This concept has been explored by Rothenberg [10], who showed that people who connected through sex transactions or shared needles with others when injecting street drugs in Colorado Springs resided 5.3 km apart on average, while those who engaged in *both* activities lived an average of 3.2 km from each other. Rothenberg also traced sexual contacts of people with gonorrhea and defined a network of people directly connected to one another through sex (a sexual network) in U.S. census tracts [11], reinforcing that small distances are a determinant of risk behavior participation among social contacts [10,12,13]. De *et al*. [14] showed that a gonorrhea outbreak in northern Alberta communities could be traced back to eighteen sexual network members who may not have had sex with each other but who all met a partner at the same bar; the bar itself created many connections in a network that may otherwise not have formed. This was further highlighted by Emch *et al*. when they demonstrated that contaminated spaces are important transmission routes for diarrheal infections in familial social connections [15]. Molecular sequencing of bacteria has also indicated that venues play a key role in the transmission of tuberculosis through the interaction of people within contaminated air spaces [16].

Spatial clustering of similar strain types of chlamydia within sexual networks has demonstrated the importance of people who bridge distinct geographic and demographic groups [8,17]. While geographic clustering of events is unsurprising in analysis of BBP and STI incidence, the dependence of their spread on interaction indicates social clustering is as important as spatial clustering. Therefore, mapping connections between individuals and places has suggested that specific venues at both short and long distances from patients’ residences are related to risk interactions among social contacts [10,12,14].

The *place* that individuals engage in risk-related behaviours may be as much a part of the network of activity as the people they connect with. This notion was explored by Cummins *et al*., who suggested that application of place to health solutions has relied too much on the conventional, Euclidean view of space in the past [18]. Instead, a relational perspective to place and distance is suggested to study landscapes of social geography. For example, two people may continually live in distant locations but their relational activity space—attending the same school for a period of time or visiting the same location while traveling—can bring them very close together. Likewise, a bath house where people meet to have unprotected, anonymous sex may be the only perceptible physical link between people who live very far away [19].

Thus, social interaction networks form transmission routes within designated spaces chosen by the participants. This forms what we term a “sociospatial network,” wherein characteristics inherent to the culture of the network are conferred on chosen locations. The places in the social interaction network serve as a means to access other people from whom drugs, drug equipment, disease prevention information, clean needles, and/or condoms may be available. It may also be a place in which used drug equipment is discarded (allowing reuse) or sex workers may be engaged. The presence of coincident yet unconnected networks involving risk-related behaviours suggests unidentified links may arise due to forgotten, unreported or anonymous connections, making it challenging to identify complete transmission routes [8,20].

Potential social connections omitted in responses to social network questionnaires may be inferred from the shared locations where these social behaviours are practiced. Our objective was to test the feasibility of using social network data together with locations of residences and risk activity places to determine whether specific areas could be defined with enough precision to infer contact between people who range from casual acquaintances to people who recognize each other only on sight. In this way, we expect to address the missing links in social networks.

## Methods

Social network data was obtained using a respondent driven sampling (RDS) of injection drug users (IDU), street youth, men who have sex with men, and homeless people in Winnipeg, Manitoba in 2009 [21]. The ethics boards of both the University of Manitoba and Queen’s University approved the study protocol. Only informed verbal consent was obtained from all participants due to high rates of illiteracy among the study population, with the interviewing nurses’ initials confirming the consent form was read aloud and explained to respondents, and that verbal consent was given. This consent procedure was approved by both ethics boards named above. Special considerations in the consent process are given to those who are under the age to consent to sex, which is 16 in Canada (under Bill C-22). Once living on their own, seeking health care for drug or alcohol use, or sexual health care for birth control, these young people are considered emancipated minors who, by the act of seeking help for themselves, are deemed to be adults and are fully able to consent. Our interviewing nurses are accustomed to working with vulnerable people and assess a participant’s state of mind while seeking consent. They do not seek consent or complete the questionnaire for a participant who is too far under the influence, nor will they conduct one when the individual is in withdrawal, which may also affect his or her mental state significantly. In these cases, the nurses have deferred the interview to a later date. In addition to this, should the individual exhibit signs of withdrawal while being interviewed, the nurses would break off and return once he or she had a dose.

Respondent driven sampling as conducted in this study has been described in depth [21]. Briefly, participants answered questions on their demographics, drug and sexual risk behaviours, social capital, neighbourhood characteristics, access to health care, social network members, and risk interactions with network members such as sex and sharing drug use equipment. Blood and urine samples were tested for HIV, Hepatitis C, chlamydia, gonorrhea, and syphilis. The specific address or nearest intersection to each participants’ current residence and the location of risk activity sites (e.g. injection drug use, sex client pickup, sex worker pickup hereafter referred to as sex trade) were reported. Casual social venues named by respondents were collected but not used in this analysis as we wanted to focus on locations of heightened risk activity and exposure to infection, where shared use of the space by network members might link them through characteristics of the site (e.g. discarded needles, unreported social interaction). Initial participants—known as “seeds”–were chosen by the research nurse based on her knowledge of the community in which she had been working for 15 years. Fifteen seeds were chosen from injection drug users, four from street youths, nine sex workers, and four men who have sex with men (MSM). Each seed was given 3 coupons with the study information and contact numbers to give to friends and family members who practiced the behaviours in the questionnaire (coupon referrals). People who received the coupons (recruits) could contact the research nurse, decide where in the community they wanted to be interviewed, give informed consent, complete the questionnaire and laboratory testing, receive the honorarium of CDN$40.00, as well as three coupons which they could give to three new friends or contacts. Individual seeds and their recruits, who in turn recruit more friends into the study, form recruitment chains of participants connected by at least one link. Although egocentric social network data of each participant were collected for up to 10 network members, these nominations did not necessarily include the person to whom the coupon was ultimately given. The nurse interviewer’s familiarity with the community prevented participants who received two coupons from two different recruiters from being interviewed twice.

Geographic locations of named intersections were provided through the questionnaire for: “What is the nearest intersection to the place you most frequently lived in the most over the last 6 months?”; “Thinking of the places above where you have injected most frequently—what is the nearest intersection to that place?”; “Thinking of the place where you most frequently meet your client partners—what is the nearest intersection to that place?”; and “Thinking of the place where you most frequently meet your sex worker partners—what is the nearest intersection to that place?” Each geographic location was verified using Open Street Map (www.openstreetmap.org) as a base layer and a point manually added to represent that location. Each point contained a latitude and longitude representing either a street intersection or place name and an identification number representing a residence, location where sex was bought/sold, or location where drugs were injected; the nurse interviewer was consulted where locations were not known to the author (JL) (M. Ormand, personal communication, January 23, 2014). Any locations that could not be verified or did not contain geographic information were not coded. We considered all identified locations part of the “risk activity space” as preliminary analysis of the data found that 15.8% of participants reported that they injected at private residences. Once coded, each location was referred to as R1 through R101, numbered sequentially after the data were randomized.

### Social Network Analysis

The RDS method applied here limited the number of recruits referred by any respondent to three; although shared locations associated with risk activity can link people not identified by RDS referrals. To create the social network, we used Pajek—an open source software developed by [22] for the analysis and visualization of large networks, used previously to graph STI-infected individuals with large sexual networks [23]. Individuals (“nodes”) in a social network form a series of “components”, where each component is made up of nodes connected through at least one link. In this case the components are representative of recruitment chains as no other social linkages were included in the research.

We performed two analyses. First we reconstructed the social network of participants to understand how participants were linked and how large these components were. This provided a baseline for which to compare the influence of locations. A one-mode, undirected network was constructed. Second, we included location when constructing the social networks. Again a one-mode, undirected network was constructed with nodes representing residential addresses of each individual and their nominated risk spaces; three locations for injecting and one location each for sex client and sex worker. Links were formed by connecting each individual’s residence to his or her risk sites and recruits’ residences.

The relative importance of locations was determined by counting the number of connections to immediately adjacent nodes (degree). Betweenness centrality, represented as:

$$C_{B}(\upsilon )=\overset{}{\underset{s\neq \upsilon \neq t\in V}{\sum }}\frac{\sigma_{st}(\upsilon )}{\sigma_{st}}$$ (1)

gives the proportion of shortest paths between any two nodes s and t on which any node υ lies [24,25]. Essentially, this allows us to quantify the likelihood that any residence or risk site lies on a short path between two other locations, providing opportunities for transmission through the sociospatial network to people who may otherwise not be connected. Betweenness was used here on the basis that each point represented a site in the network model. We selected this measure over others because it captures the conscious tendency of people to take the shortest routes between their own residences, friends’ residences, and risk sites [26]. In our application, it defines the likelihood of exposure to a ‘risk activity location’ either by meeting outside a residence/site en route to other locations and may be a function of housing choice, social role of the individual, or both. Whichever the case, it captures social associations and interactions through which infections may spread.

### Spatial Analysis

The integration of social network analysis with spatial analysis was used to illustrate the social topology present among respondent networks. The outputs were used to understand the spatial concentration of network components and the distribution of respondents with respect to where the social topology was strongest. ArcGIS 10.2 [27] was used to map the activity space of each of the largest components identified through the social network analysis by constructing a convex hull. Kernel density estimation (KDE) was used here to infer areas of risk behaviour where spatial clusters are coincident using high social network betweenness derived from the social network analysis. The KDE function fits a surface over each point in the dataset, using the points or the values of the points within a chosen radius, here defined as 500 metres to represent one large city block in Winnipeg. In this way it defines a space within which certain areas are more “risky” than others; or more likely to be the venue for high risk activities. KDE is well suited to this analysis as it allows for the fact that two people may not be present concurrently but allows for the possibility of exposure through risk activities (ie. contact with contaminated, discarded injected drug equipment) at a place they both visit. Thus, it captures overlapping areas visited by each individual. We first examined the point locations to identify areas nominated frequently through responses. A second application of KDE assigned the betweenness score of each point, aimed at highlighting locations of greatest influence.

## Results

The study population of 600 individuals was 52.8% male, ranged in age from 14–78 years, and 73.3% were of North American Indian ancestry. Over half of respondents stated they had injected drugs (50.5%) but only 16.2% of those provided an identifiable geographic location in which they injected drugs. All but two IDU had injected in the past 6 months; 47.2% reported sexual contact with someone outside of their close social network; and 10.2% had sex with at least one sex trade partner in the same period. Fifty-one respondents who bought or sold sex provided a response as to where they most frequently met those partners. Serological testing for STIs and BBPs was refused by 13.2% of respondents. Of those who consented to tests, laboratory results showed 8.0% infected with HIV; 28.2% infected with Hepatitis C (HCV); 3.5% with chlamydia; 0.8% with syphilis.

Overall, 93.6% (719 of 768) of locations nominated by respondents were successfully identified and geocoded including residences (93.2%), IDU sites (85.1%), commercial sex client recruitment sites (84.8%), and sex workers recruitment sites (87.5%). The remaining locations could not be geocoded due to uninterpretable spellings, naming of two parallel streets, or nonspecific designations; thirteen individuals did not provide any response regarding their residence. One hundred and one locations were identified as unique risk activity sites (IDU or sex trade).

**Fig 1:**
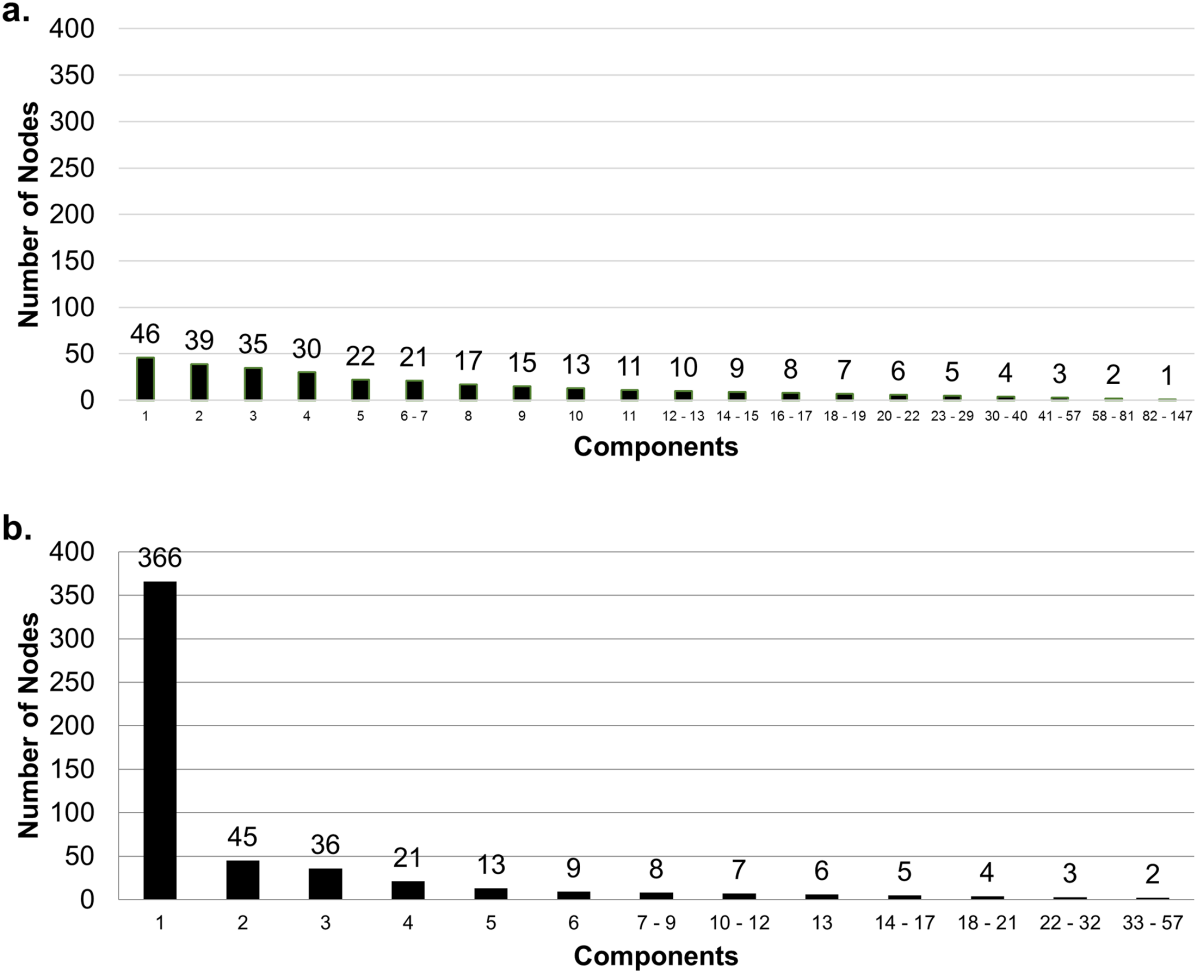
Number of social networks (a) without geography and (b) in the sociospatial model. (A) shows social network size by number of respondents connected, before including sociospatial risk sites (n = 600, N = 147), (B) captures the **s**ocial network size by number of nodes (connected respondents and risk sites) in the sociospatial model (n = 701, N = 57).

**Fig 2:**
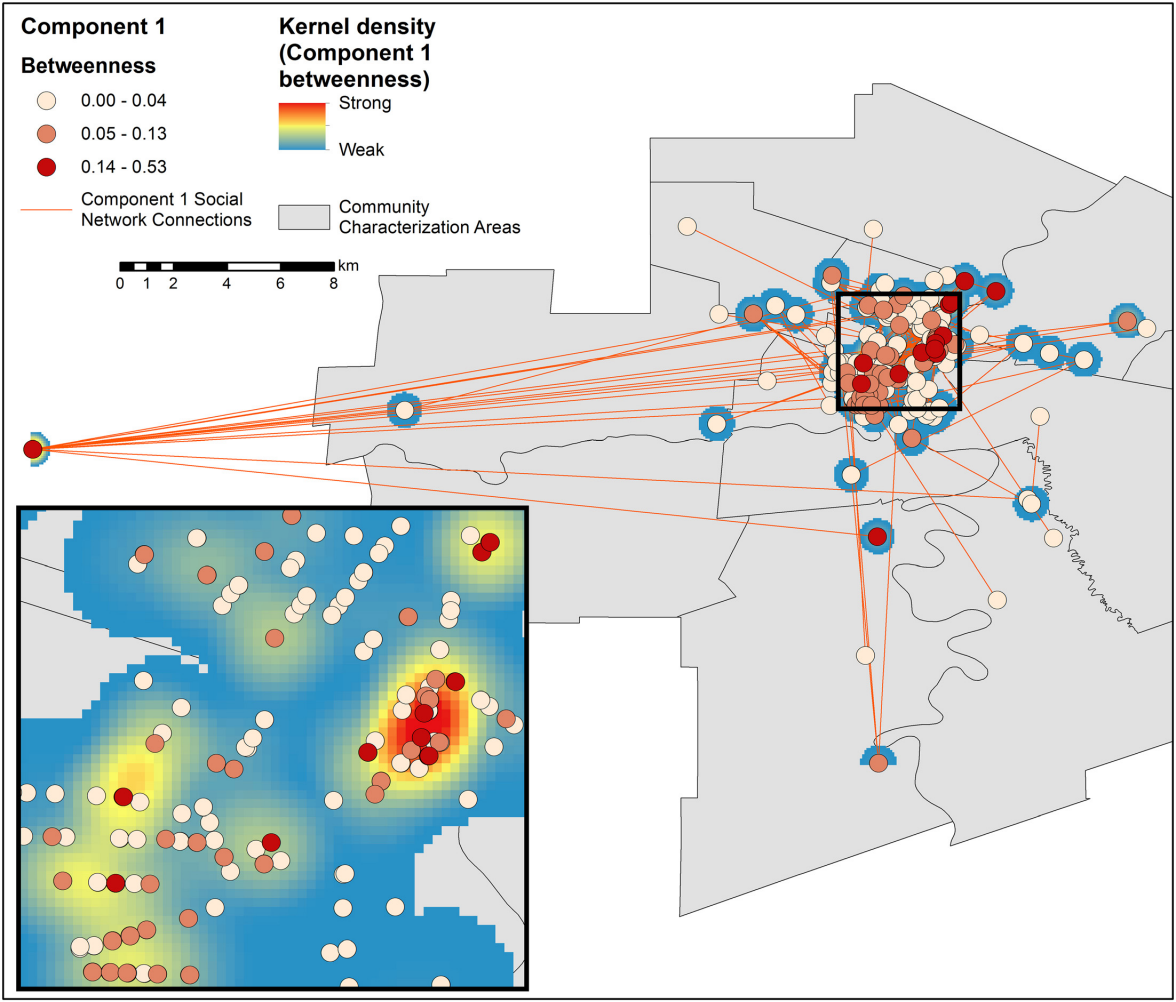
Geographic extents of Component 1 (n = 366). Points, demonstrating spatial distribution of residences and risk activity sites, are colored by betweenness centrality scores. The KDE surface for Component 1 is calculated by weighting the betweenness scores in the sociospatial network. (Created using ArcGIS 10.2 [27]. Community Characterization Area base layer obtained from the City of Winnipeg’s Open Data Catalog [28].)

**Fig 3:**
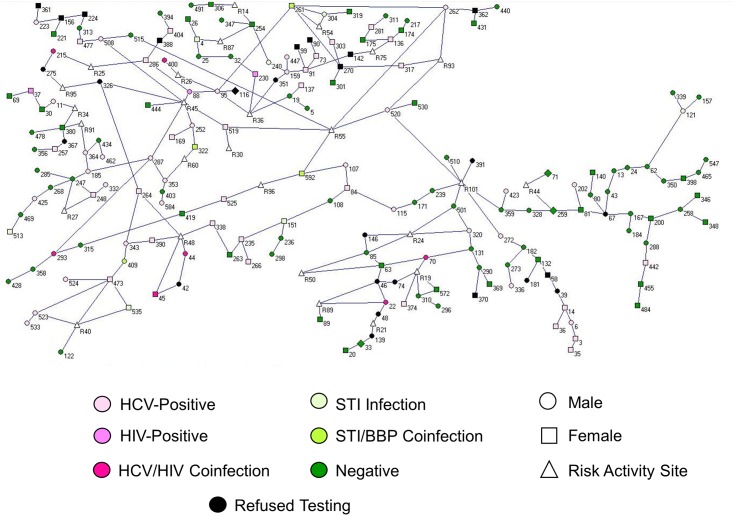
Trimmed network graph of sociospatial Component 1 (n = 366). This network graph of the largest sociospatial network component is trimmed to isolate highly-connected nodes. Symbol shape and color illustrate gender, infection status, and risk activity sites. (Created using Pajek [22].)

The sample was segmented into 147 separate components (Fig 1a), the largest of which was 46 individuals, accounting for 7.7% of the sample. When place was used to represent individuals and their activity sites in the sociospatial network, the largest component included 49.7% of all nodes (Fig 1b). Fifty-seven components were identified in the sociospatial network, with no isolates (unconnected nodes). The largest contained 366 nodes (Figs 2 and 3). The infection status and gender of participants as well as their connection to other participants and the risk sites they have named is illustrated in Fig 3. Although we have omitted all nodes with only one connection to the component for visual clarity, the high connectedness and variety of people with sexual and drug-using risks and infections is clear within this single connected group. This is demonstrated in the connections made through site R48 (sex trade and IDU), which link five otherwise unconnected people (three with HCV and two with a HCV/HIV coinfection).

The composition of the sociospatial network also included 43.9% components containing only two nodes (dyads); 19.3% connected three nodes (triads); five components were greater than size ten. Twenty-three components (40.4%) included at least one geographic location, of which the largest contained over two-thirds (67.3%) of the risk spaces. Component 7 (n = 13) did not have a single risk-related activity site among its nodes. Twenty-eight percent of dyads comprised one respondent linked to a location but no other people, while 36.4% of triads included one geographic place and two respondents.

**Fig 4:**
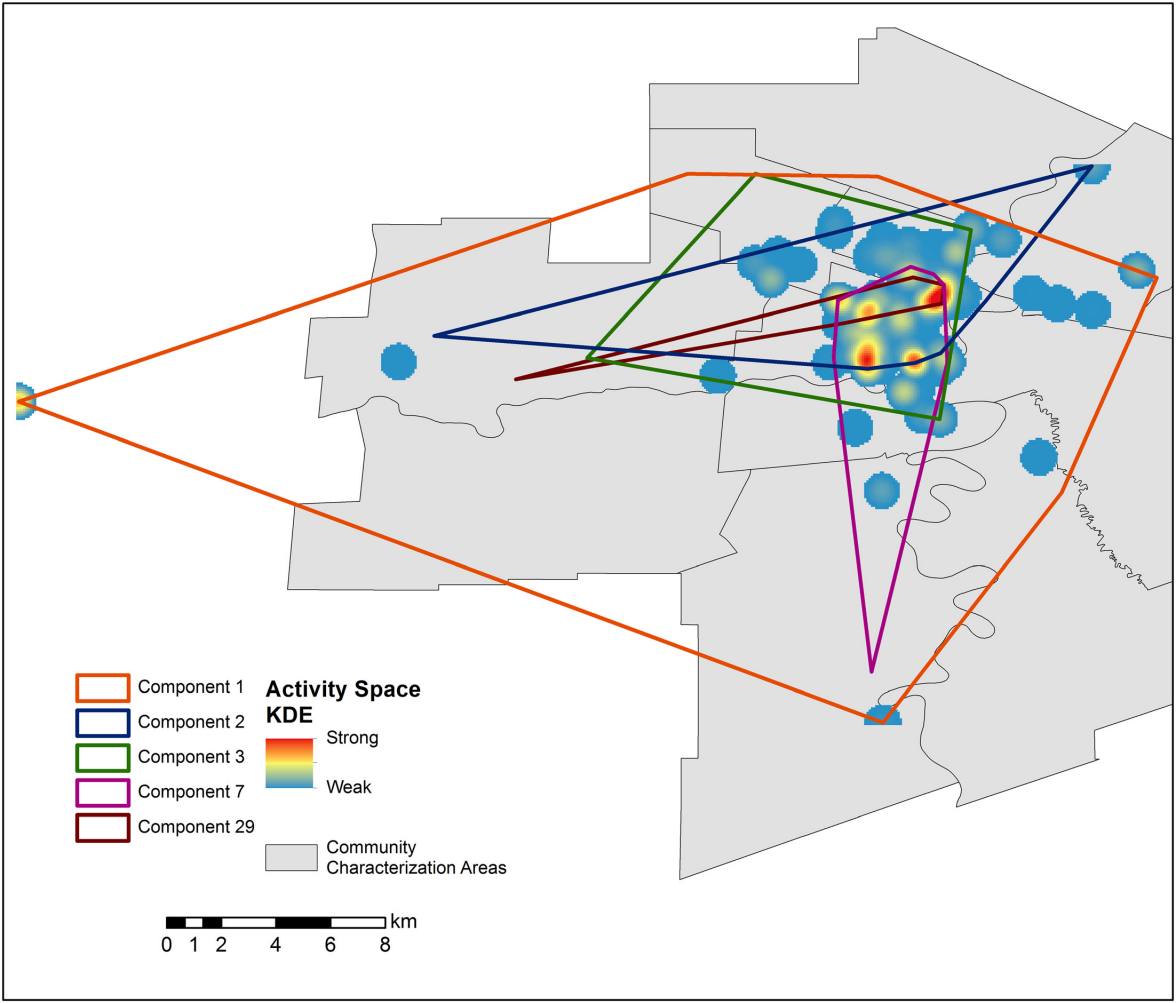
Spatial extents of the five largest sociospatial network components. The intersection of components 1 (orange), 2 (blue), 3 (green), 7 (purple) and 29 (brown) and a density ‘heat map’ of all risk activity locations illustrates where each component presents risk. (Created using ArcGIS 10.2 [27]. Community Characterization Area base layer obtained from the City of Winnipeg’s Open Data Catalog [28].)

The five largest components (Fig 4) account for 72.9% of the nodes in the analysis. Twenty-two percent of connections were less than 100 metres, and forty-four percent were less than a kilometre apart. Table 1 shows key epidemiological characteristics for the largest components with Component 1 exemplifying the whole sample. The high “unknown” rates denote the proportion of the study sample who did not report risk locations. The mean distance of the five largest components was within 3.5 kilometres between any two connected respondents’ residences or a residence and a risk activity site. Risk spaces for each of the major components are highlighted in red (highest), yellow and blue (lower), indicating their social importance (Figs 2 and 4). Several pockets of activity occurred throughout the city, with individual sites such as R101, at the western extent, R45 and R55 in downtown Winnipeg as foci of activity. The densest areas of social activity fall within the overlap of all five components, coinciding with the core of Winnipeg (Fig 4). R101, which is a penitentiary outside the city’s western limits, had the highest betweenness score and strongest social influence in Component 1. The remaining nine highest scores were composed of seven residential addresses of people and two other risk sites. R45 (sex trade and IDU) and R55 (IDU only) together form the areas named most (highest degree centrality) and with the highest betweenness-weighted density for Component 1. For the remaining components, only two risk-related activity sites were identified with betweenness values higher than zero: R5 (injected drug use, Component 3) and R31 (sex trade, Component 29).

**Table 1:** Characteristics of participants in largest components of the sociospatial network.

Component	Gende*r (% Male; % Female; % Other)*	Risk Activity *(% IDU; % Sex Trade; % Both; % Unknown)*	Disease* *(% with STI; % with BBP; % Both; % Refused)*	Ethnicity *(% Caucasian; % First Nations; % Métis; % Other)*	Mean Distance (+/- SE) (km); Max Distance (km); Component Area (km^2^)
**1 (*298 people*, *68 risk sites)***	56.4	21.5	4.7	20.8	3.6 (+/- 0.02)
	43.3	6.0	32.6	56.4	27.3
	0.3	3.0	13.1	19.5	296.1
		69.5	0.7	3.3	
**2 (*43 people*, *2 risk sites)***	55.8	4.7	2.4	44.2	2.1(+/- 0.09)
	44.2	0.0	41.9	27.9	16.7
	0.0	0.0	11.6	18.6	46.5
		95.3	0.0	9.3	
**3 (*32 people*, *4 risk sites)***	46.9	15.6	3.1	56.3	3.1 (+/- 0.10)
	53.1	0.0	28.1	28.1	10.2
	0.0	0.0	3.1	15.6	51.0
		84.4	3.1	0.0	
**7 (*21 people*, *0 risk sites)***	61.9	0.0	0.0	23.8	3.0 (+/- 0.26)
	38.1	0.0	9.5	52.4	14.9
	0.0	0.0	9.5	9.5	22.9
		100	0.0	14.3	
**29 (*11 people*, *2 risk sites)***	54.5	9.1	9.1	27.2	2.6 (+/- 0.43)
	45.5	18.2	27.3	36.4	10.7
	0.0	0.0	9.1	18.2	6.4
		72.7	0.0	18.2	

Characteristics of components 1, 2, 3, 7 and 29 represent the answers provided by respondents. Percentages are calculated based on the number of participants in each component. Spatial characteristics of each component indicate the average Cartesian distance between two nodes in that component as well as how much area each covers.

* Disease status will not total to 100%.

## Discussion

We demonstrate here that spatial analysis using social network metrics can be used to better understand the social topology that enables STI/BBP transmission among IDUs and sex trade participants. We found that risk activities were highly concentrated in certain neighbourhoods, as has been shown in other cities [29], reflecting the compact spatial nature of the majority of the social connections. The importance of location in risk activities is supported directly by two independent phenomena: the re-use of contaminated injected drug equipment, which may be discarded in these risk activity spaces and surroundings, and by the organisation of sex worker strolls or territories by street or street corner, either by their own agreement, or enforced by pimps. Less direct exposures may include inviting passers-by to purchase sex or drugs.

**Fig 5:**
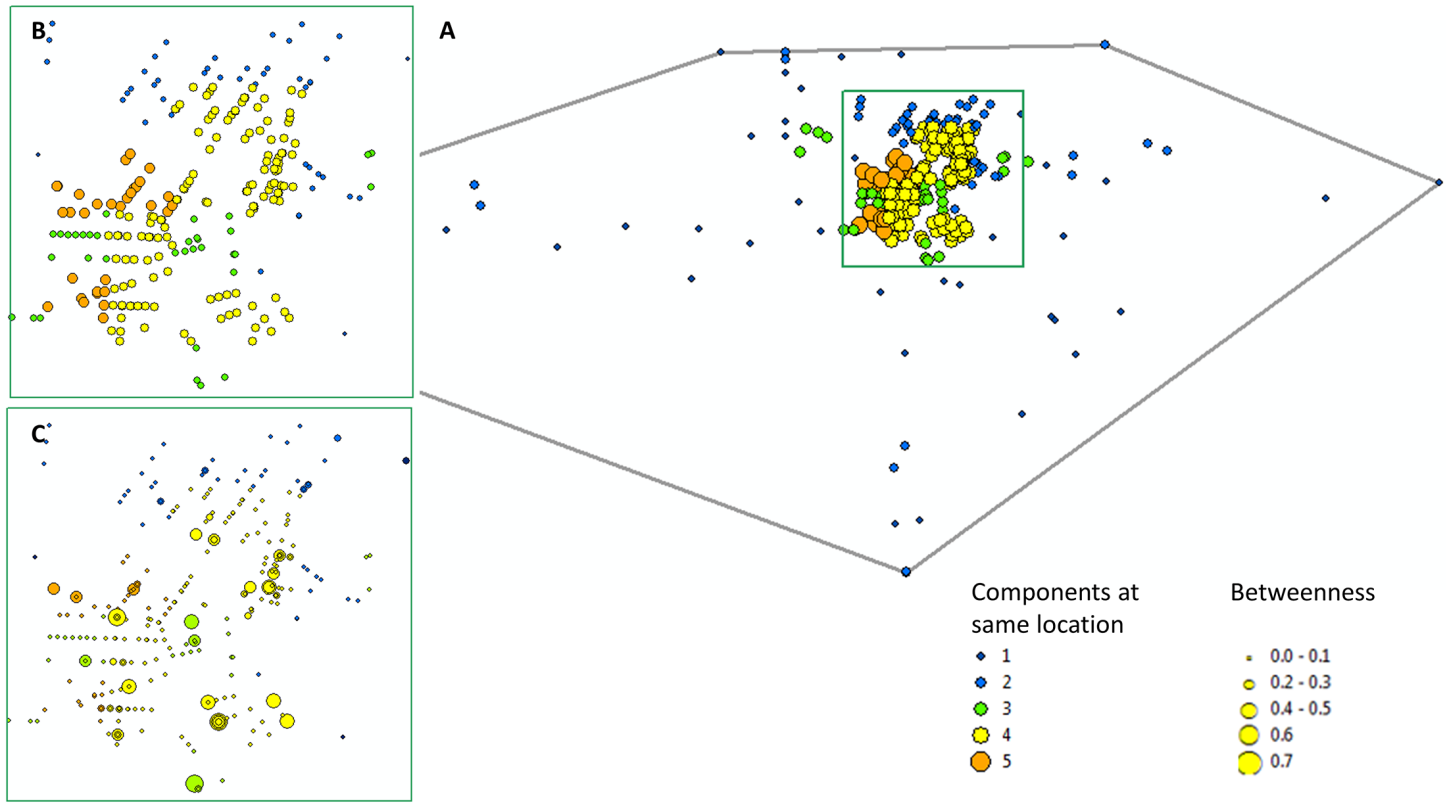
Spatial co-incidence of components. Spatial co-incidence of components for the entire study area (A) represents the spatial distribution of the entire sociospatial network. (B) highlights the number of places frequented by each of the five components and (C) the betweenness of each location for each of the five components.

The inclusion of the risk activity sites demonstrated a much more cohesive network than that identified by person to person connections alone. Without the strong influence of several sites (R45, R55 and R101 in particular), the network was split into a high number of isolated components (Fig 1a). By taking place into account we were able to show the overlap of the different social network components with risk venues, capturing the number of components that interact at the same location. For example, in Fig 5 we show the number of components for which members interacted at the same location. Although these components appear to be separate social networks they can be found occupying the same places. This close geographic proximity (within 3.5 km) suggests that people in the other large components may also be linked to Component 1.

It is notable that through the sites of risk-related behaviours for 7.7% of the people in this study, 49.7% of the high-risk people could be accessed. This is the greatest difference between a small group of selected people leading to a large sexual and injection drug use risk network found to date, the second highest being a gonorrhoea outbreak in Alberta in which 49% of the network was accessed through 21% of individuals [14]. In a similar analysis of an outbreak of 29 people infected with the same strain of tuberculosis, the largest connected component initially included only 5 (17.2%) people. While a number of places connected some of the participants, the most frequently patronized establishment was a bar visited by local workers in the afternoons and early evenings and used as a popular location for gay men to meet sex partners at night. In this case, all 29 people with tuberculosis could be connected through locations. Many of the men did not know each other, but breathed contaminated air in the bar [16]. This example demonstrates the potential for spaces to become “carriers” of infection. It is noteworthy that for vulnerable street populations and particularly Canadian First Nations people, tuberculosis is a significant health problem [30].

Implications of this work are far reaching. First, one of the key assumptions of RDS inference is that a single network component is being sampled [31–35]. These results suggest that this indeed may be the case, which would simplify interpretation of RDS estimators. Second, prevention services delivered by community health care workers on foot or in vans, such as point of care testing, needle distribution, and further research, can be targeted at smaller areas of the community or within selected groups to reach a high proportion of people at risk. Third, through the intensity of connected social activity concentrated in only a few locations (Figs 2 and 3), we see additional evidence that social networks of vulnerable populations exhibit a small world network structure, where a small number of risk activity sites play a role similar to that of airport hubs [36]. These types of networks have specific properties which foster disease spread, and resist prevention with randomly selected participants [37]. Fourth, these results imply that time-space sampling may capture a larger proportion of the target populations than previously thought, while more clearly identifying those who may be missed. Fifth, the importance of institutions such as penitentiaries is emphasized here, despite their relatively long distance away from the central hubs of activity. This emphasis is all the more important given the gaps and challenges in applied research and interventions within these institutions. Last, and crucially, these risk spaces, when enclosed, are clear contenders for the transmission of other diseases, such as tuberculosis [16].

There are limitations of this study that include an underestimation of links due to underreporting of people and places (numbers of coupons and place names were each limited to three); underreporting of personal risk activities [38]; and underestimation of the size of the risk spaces [39]. The fact that many people chose not to name intersections where they participated in commercial sex work or injected drugs is understandable. The information could be used by police to target and harass street people. In cases where participants have injected in friends’ homes, interviewers may encounter reluctance to implicate those social contacts. It is also possible that individuals only inject in their own homes. In these cases, missing data leads to an underestimation of network size and numbers of common locations, leading to a less cohesive structure than the true measure. It is also impossible to determine exactly errors associated with the identification of the “nearest intersection”. Two participants may name different intersections one street apart from each other. They may be nominating two mutually exclusive risk sites, yet it is possible they may each identify the same site differently.

However, despite these limitations, the exploratory approach defined a geographically and socially cohesive community through which infections spread. Previous ecological studies and data on distances between infected people [10,12,39] suggested its existence. Here, we anchored the social interactions in geographic space resulting in small activity spaces containing people with different demographies, infections, and risk behaviours. Not only were we still able to detect cohesiveness expected in the data but also identify key risk activity locations and the interaction of these with overlapping activity spaces of each of the components identified. What we may very well lack are nominations of less common and more widely dispersed points which may form smaller components away from the largest one we have examined here. Future studies will elicit more detailed information about the geography (through the identification of locations using recognizable features such as landmarks [29] or the use of smartphone GPS technology) and the time aspect of risk activities. The use of spatial analysis methods such as KDE to model areas of risk could also benefit from exploration into cell size, adjusted to reflect practicable distances of areas of influence (e.g. distance to recognize a familiar person, shouting distance).
